# Our New York Letter

**Published:** 1886-03

**Authors:** Wm. Penny

**Affiliations:** New York


### OUR NEW YORK LETTER.

### GYNECOLOGICAL NOTES FROM THE N. Y. HOSPITALS AND POLYCLINIC.

NEW YORK, Feb. 17, 1886.

ED. DANIEL'S MEDICAL JOURNAL :

ACCORDING to promise, I will give you a few notes on Gynecology in New York. I shall treat the subject in a general way, leaving description of special operations for some future time. My reason for this is, that there are no radically new operations in this branch of surgery, and the operations in vogue have been described with such minuteness of detail, by Thomas and Emmet, as to leave little or nothing for a letter writer.

In my desire to give your readers something entertaining, and wishing to be instructed myself, I attended a meeting of the Academy of Medicine, to listen to the reading of a paper on

"Free Drainage of Pelvic Abscesses," by a professor of gynecology, and to the discussion of the same.

There are but few subjects of greater interest to the general practitioner than this, and I naturally expected to learn a great deal that was new, at least so far as concerned the surgical treatment of this supposed to be formidable disease.

Imagine my surprise, when I listened to this essay. The essayist frankly admitted in the course of the discussion that there was nothing new in it. But there was something new in it after all, for the essayist alluded to the case of Dr. Polk, where he tried the external illiac artery to gain access to a small pelvic abscess; an operation that has been repeated in the journals, and criticised favorably and otherwise, but always with credit to the boldness of the operation.

There was something else that was new, in spite of the refreshingly candid denial of the essayist. That was, that in his extensive practice, he had never had a case to terminate fatally. After reading Simpson, Thompson, Tait, et omne generis, this candid admission bears on its face the true stamp of modesty; otherwise it might be inferred that the essay was written for the opportunity of saying just this, and nothing more.

In the course of the discussion, one of the members gave the history of a case in which an abscess was produced, or if one existed, it was made greatly worse by efforts at aspiration, and this would hardly be worth relating, were it not that this member has since publicly confessed that the apostle of free drainage was the consultant.

Another professor took up the subject from a different standpoint; that of difficulty in diagnosis. He said that he had under observation two cases that he was in the habit of bringing before his class as typical cases of ovarian tumor. They had all the rational and physical signs, but to his surprise they both proved to be cases of pelvic abscess by opening spontaneously. The professor could not omit the opportunity of having a backhanded lick at the country practitioner. He related an incident where a country practitioner said to him that, so far as his observation went, cases of pelvic abscess always terminated fatally. Now, this is one of the little playful methods by which we let the provincial brother know his inferiority. In fact, we gynecologists feel that we occupy a position like the priests of old, that we are a profession set apart to prevent the female portion of the population from going to utter destruction, and nothing but our excessive modesty prevents our letting this be known.

Do not infer from this levity that there are no earnest workers in this field. The Titans still remain, except the master spirit, that has departed. The field has been closely harvested, but with the inevitable result of over crowding this specialty; and we find a large number of young men who, waiting for the mantles of the great to fall on them, have grown gray waiting.

Of operations there are the usual ones. Ovariotomies are, as a rule, performed earlier than formerly, and the result is that they are not so showy as formerly. Tait's operation is having a run, if I may use a theatrical expression. It has been my misfortune not to have seen what I would call a diseased tube removed in the operations that I have seen performed.

Emmet's operation for lacerated cervix, as you know, has found its legitimate position, and is performed quite frequently. Alexander's operation has not met with much favor here, and in fact, it is applicable to an extremely limited number of cases. Emmet's operation for lacerated perineum is frequently performed. It is somewhat difficult to perform, and still more to describe.

All these operations, so far as can be done, are performed under strict antiseptic precautions. Where this is not practicable antiseptic dressings are used. WM. PENNY, M. D.

